# Predictive value of baseline levels and early dynamic changes of ctDNA for 28-day mortality in ICU patients with advanced lung cancer: a prospective single-center cohort study

**DOI:** 10.3389/fmolb.2026.1811387

**Published:** 2026-06-23

**Authors:** Liping Tan, Dongxi Lu, Haijiao Huang, Dongyi Lin, Dingwen Zheng

**Affiliations:** 1 Department of Respiratory Oncology, Guangxi Medical University Cancer Hospital, Nanning, Guangxi, China; 2 School of Public Health, Guangxi Medical University, Nanning, Guangxi, China; 3 Department of Traditional Chinese Medicine, Guangxi Medical University Cancer Hospital, Nanning, Guangxi, China; 4 Department of Cardiac Surgery, Sir Run Run Shaw Hospital, School of Medicine, Zhejiang University, Hangzhou, Zhejiang, China

**Keywords:** cancer biomarker, circulating tumor DNA, intensive care, lung cancer, prognostic model

## Abstract

**Background:**

Critically ill patients with advanced lung cancer admitted to the ICU have high short-term mortality and heterogeneous outcomes. Conventional severity measures reflect organ failure but not tumor-related activity.

**Objective:**

To assess whether baseline ctDNA and 72-h ctDNA dynamics are associated with 28-day mortality and whether they add incremental prognostic value beyond clinical variables.

**Methods:**

In this prospective single-center cohort, 293 patients formed the full cohort; a 72 ± 6-h landmark dynamic cohort included 233 patients alive and evaluable at 72 h. Plasma ctDNA was tested at ICU admission and 72 h. Multivariable logistic regression and restricted cubic splines assessed associations. Model discrimination, calibration metrics, reclassification, and decision curve net benefit were compared before and after adding ctDNA.

**Results:**

Baseline ctDNA was higher in non-survivors than survivors (P < 0.001). Baseline ctDNA [ln(VAFmax_T0+0.05)] was independently associated with 28-day mortality and showed a nonlinear relationship (Poverall<0.001, Pnonlinear = 0.028). In the landmark cohort, ΔctDNA was independently associated with mortality from 72 h to day 28 (adjusted ORs 1.86 and 1.62; both P < 0.01). Adding ctDNA improved performance: AUC increased from 0.78 to 0.84 and Brier score decreased from 0.201 to 0.184 in the full cohort; AUC increased from 0.75 to 0.80 and Brier score decreased from 0.219 to 0.207 in the dynamic cohort. NRI/IDI were 0.42/0.08 and 0.29/0.05, respectively.

**Conclusion:**

Baseline ctDNA and early ctDNA dynamics were independently associated with short-term mortality and modestly improved prognostic model performance. These findings suggest potential value for ICU risk stratification pending external validation.

## Introduction

Lung cancer remains one of the leading causes of cancer-related death worldwide, with a high proportion of patients at an advanced stage, and patients often require intensive care after developing infection, respiratory failure, or shock (Andréjak et al., 2011). Critically ill patients with advanced lung cancer admitted to the ICU often have multiple organ dysfunction, a short window for treatment decision-making, and highly heterogeneous prognosis; clinically, early risk stratification after ICU admission is needed to support the intensity of organ support, communicate prognosis, and allocate resources (Qian et al., 2023; Elkhapery et al., 2025). Current assessments mainly rely on physiological indicators such as SOFA, APACHE II, and lactate, which can reflect the severity of organ failure but have difficulty quantifying the contribution of tumor burden and tumor biological activity to short-term mortality (Song et al., 2019; López et al., 2021). Although imaging staging and prior treatment history are important, it is difficult to characterize the current tumor activity and reversibility of the disease course during the acute critical stage (Buchtele and Munshi, 2025). Repeatedly measurable blood biomarkers are expected to provide Supplementary Material without increasing invasive burden. Circulating tumor DNA can carry tumor-specific variants and quantitatively reflect tumor burden and cell turnover, and it is cleared relatively rapidly in plasma, theoretically making it suitable for capturing changes in condition on the scale of hours to days (Kim and Park, 2023). Existing evidence mainly comes from outpatient oncology and systemic treatment settings, with endpoints mostly being efficacy evaluation or long-term survival (Ricciuti et al., 2021); studies targeting critically ill lung cancer patients in the ICU are scarce (Park et al., 2021). This population often has impaired circulatory perfusion, compromised hepatic and renal function, and a strong inflammatory response, which may affect ctDNA release and clearance, so its performance in short-term prognosis cannot be simply extrapolated (Kustanovich et al., 2019). More importantly, there is still a lack of prospective cohorts that simultaneously evaluate the independent predictive effect of baseline ctDNA at ICU admission and early dynamic changes within 72 h on 28-day mortality, and there is also a lack of systematic validation of its incremental predictive value, reclassification ability, and clinical net benefit beyond traditional variables such as SOFA, lactate, and organ support. A single-center prospective cohort was established, enrolling critically ill patients with advanced lung cancer admitted to the ICU; baseline plasma was collected within 6 h of ICU admission and retested at 72 ± 6 h, and plasma ctDNA was tested. The study evaluated the independent predictive effect of baseline levels and early dynamic changes on 28-day all-cause mortality, and compared model discrimination, calibration, and decision curve net benefit before and after adding ctDNA. The study verified that ctDNA can provide incremental predictive information beyond traditional critical illness variables.

## Materials and methods

### Study design

This study was a prospective single-center cohort study conducted in the general Department of Critical Care Medicine of a tertiary teaching hospital. The enrollment period was from 1 June 2022 to 31 May 2024, and follow-up continued until 31 May 2025. The study aimed to evaluate the predictive value of baseline ctDNA levels at ICU admission and early dynamic changes in ctDNA after ICU admission for 28-day all-cause mortality, and to assess the incremental predictive ability of ctDNA on the basis of routine clinical variables and critical illness scores. During the study period, ctDNA results were not returned to the clinical team and were not used for diagnostic and treatment decision-making. The study protocol was reviewed and approved by the hospital ethics committee. All patients provided written informed consent before enrollment.

### Study participants

The study participants were consecutively admitted critically ill patients with advanced lung cancer admitted to the ICU. Advanced lung cancer was defined according to the AJCC 8th edition: NSCLC stage IIIB–IV or extensive-stage SCLC (Tseng et al., 2020). Critical illness was defined as acute organ dysfunction occurring within 24 h after ICU admission and requiring organ support (meeting any of the following: invasive mechanical ventilation, non-invasive ventilation, high-flow nasal cannula oxygen therapy〔≥40 L/min and FiO_2_≥0.40〕, continuous infusion of vasoactive drugs >1 h, continuous renal replacement therapy, or intermittent hemodialysis); these thresholds were used as the prespecified operational definition of critical illness in this study (Sevransky et al., 2021). Each patient was included only for the first ICU admission event, and the baseline time was the ICU admission registration time.

Inclusion criteria: age ≥18 years; meeting the definitions of advanced lung cancer and critical illness; completion of baseline blood sampling within 6 h after ICU admission; written informed consent obtained from the patient or legal representative. Exclusion criteria: a limitation of treatment decision formed within 6 h after ICU admission(e.g., a decision not to initiate invasive mechanical ventilation, escalation of vasoactive support, renal replacement therapy, or cardiopulmonary resuscitation); concomitant hematologic malignancy or other active malignancy within the past 5 years; pregnancy; unqualified baseline blood sample; baseline ctDNA testing failure and inability to repeat sampling within 24 h.

### Blood sample collection, processing, and ctDNA testing workflow

Blood samples were collected at two fixed time points: baseline blood sampling was completed within 6 h after ICU admission; early retesting was completed at 72 h (72 ± 6 h) after ICU admission. Patients transferred out of the ICU within 72 h but still hospitalized were retested in the ward according to the window period; patients who died within 72 h only had baseline samples retained and were included in the baseline analysis. At each time point, 10 mL of peripheral venous blood was collected (K2-EDTA tubes), gently inverted 8 times after collection, the blood draw time was recorded, and plasma separation was completed within 2 h. Samples not processed within 2 h were considered pre-analytically unacceptable; repeat sampling was performed within the baseline window when feasible, otherwise they were handled as unqualified baseline blood samples. Plasma was processed by two-step centrifugation (1,600 g × 10 min, 4 °C; after transferring the supernatant, second centrifugation at 16,000 g × 10 min, 4 °C), aliquoted into DNase-free cryovials (1.0 mL/vial), and stored at −80 °C. The buffy coat was preserved simultaneously for paired WBC DNA sequencing to remove germline variants and clonal hematopoiesis-related variants.

ctDNA extraction used the QIAamp Circulating Nucleic Acid Kit (Qiagen), with 4 mL of plasma input per sample and elution in 50 μL; DNA quantification used the Qubit dsDNA HS Assay (Thermo Fisher Scientific) (Qiagen, 2019). For library preparation, 25 ng of cfDNA was used whenever available; when the extracted amount was <25 ng, all available cfDNA was used. Library preparation used the NEBNext Direct® Custom Ready Panels kit (New England Biolabs, Ipswich, MA, United States) with a 12 bp UMI system, and PCR was fixed at 12 cycles; targeted capture used a custom-designed 168-gene panel based on NEBNext Direct® Custom Ready Panels (New England Biolabs, Ipswich, MA, United States), covering common lung cancer driver genes and clonal hematopoiesis-related genes (covering all exons and defined hotspot sites) (Jin et al., 2024). The panel was selected to cover common lung cancer driver alterations and to facilitate clonal hematopoiesis filtering; representative genes included EGFR, KRAS, BRAF, ALK, ROS1, MET, ERBB2, TP53, RB1, STK11, KEAP1, DNMT3A, TET2, ASXL1, and JAK2, and the complete gene list is provided in Supplementary Table S1. Target enrichment was performed according to the manufacturer’s instructions using a hybridization-based capture workflow, including probe-target hybridization, streptavidin bead capture, off-target sequence removal, and post-capture amplification. The sequencing platform was Illumina NextSeq 550 (PE150), with a target of mean effective depth ≥100,00× per sample after UMI deduplication.

The bioinformatics pipeline was completed in a Linux environment: after adapter trimming and removal of low-quality bases, reads were aligned to GRCh37 using BWA-MEM; consensus sequences were generated based on UMI clustering to reduce errors; Mutect2 and VarDict were used for somatic variant calling, and only SNVs and small Indels were included. Somatic variant thresholds were: UMI family count at the site ≥3, number of consensus molecules supporting the mutation ≥3, total consensus molecules ≥30, and VAF ≥0.10% (Mizuno et al., 2019). These thresholds were prespecified based on published filtering criteria for low-frequency cfDNA variants and were applied uniformly in conjunction with the study’s positive/negative controls and batch-level QC framework. Germline variants were removed if VAF in paired WBC DNA was ≥30% or the gnomAD population allele frequency was ≥0.10%; clonal hematopoiesis-related variants were removed if VAF in WBC DNA was ≥1.0% and matched the same site in plasma. Each batch included a negative control (plasma from healthy donors) and a positive control (Mimix™ Multiplex I, cfDNA Reference Standard Set, HD780, Horizon Discovery, Cambridge, United Kingdom); the negative control must not have any somatic variants meeting the threshold, and the VAF deviation of preset sites in the positive control was controlled within ±20% of the reference value. Sequencing quality control failure was defined as mean effective depth <3,000× after UMI deduplication or contamination assessment value > 1.0%; failed samples were re-prepared and sequenced once, and those still failing were deemed testing failures.

### Clinical data collection and variable definitions

Clinical data were prospectively collected by investigators from the electronic medical record and ICU information system, and were uniformly defined and coded according to a pre-specified data dictionary. Demographic variables included age, sex, body mass index, and smoking history (never/former/current). Oncologic variables included histologic type (non-small cell lung cancer/small cell lung cancer), stage (IIIB, IIIC, stage IV, or extensive stage), main metastatic sites (brain, bone, liver, adrenal gland, pleura), types of anti-tumor treatment within 30 days before ICU admission (cytotoxic chemotherapy, targeted therapy, immune checkpoint inhibitor therapy, radiotherapy), and the most recent ECOG performance status score within 30 days before ICU admission (Oken et al., 1982). Comorbidities were assessed using the Charlson comorbidity index. Reasons for ICU admission were categorized by the primary diagnosis as: acute respiratory failure, septic shock, non-septic shock, severe pulmonary infection without shock, acute central nervous system events, acute kidney injury requiring renal replacement therapy, perioperative monitoring, and others.

Critical illness-related variables were defined using the worst recorded values within 24 h after ICU admission. Critical illness scores included SOFA and APACHE II; physiological and laboratory indicators included mean arterial pressure, heart rate, respiratory rate, body temperature, arterial blood gases (PaO_2_, FiO_2_, PaCO_2_, pH), blood lactate, serum creatinine, total bilirubin, platelet count, international normalized ratio, and serum albumin. Organ support treatments recorded whether invasive mechanical ventilation, non-invasive ventilation, high-flow nasal cannula oxygen therapy, continuous infusion of vasoactive drugs, and renal replacement therapy were used within 24 h after ICU admission. Sepsis and septic shock were defined according to Sepsis-3 (Singer et al., 2016): sepsis was suspected or confirmed infection accompanied by an increase in SOFA score of ≥2 points from baseline; septic shock was defined as requiring vasoactive drugs to maintain mean arterial pressure ≥65 mmHg after adequate fluid resuscitation and blood lactate >2 mmol/L.

ctDNA exposure variables were constructed based on somatic variant results. For each plasma sample, VAFmax was calculated (the maximum VAF among all included somatic variant sites, expressed as %); for samples with no detected variants, VAFmax was recorded as 0. Given the large variation in the number of detected somatic variants across samples, and because meanVAF or medianVAF is more susceptible to low-frequency variants and the number of detected sites, VAFmax was prespecified as the primary sample-level ctDNA metric to capture the dominant tumor signal; however, because it is driven by the single highest-VAF site, it may be more sensitive to outlying high-VAF variants and may not fully represent overall tumor burden or clonal composition. Baseline ctDNA was VAFmax at T0. Early dynamic change was defined as ΔctDNA = ln(VAFmax_T1+0.05)−ln(VAFmax_T0+0.05), where 0.05 is 1/2 of the detection limit of 0.10%, used to handle non-detection and maintain transformation consistency (Hornung and Reed, 1990). For stratified presentation, according to R=(VAFmax_T1+0.05)/(VAFmax_T0+0.05), patients were classified as decreased (R ≤ 0.50), stable (0.50 < R < 2.00), and increased (R ≥ 2.00).

## Outcomes and follow-up methods

The primary outcome was 28-day all-cause mortality, defined as death from any cause within 28 days from the ICU admission registration time. Survival status was determined sequentially through the hospital information system for in-hospital outcomes, post-discharge telephone follow-up, and verification with the resident death information system. Telephone follow-up was conducted on day 28 (allowing days 27–29), with three calls made to each patient at different time periods with intervals ≥8 h; for those still unreachable, death records within 28 days were verified using the resident death information system. For deaths, the date and location of death (in-hospital/out-of-hospital) were recorded.

### Statistical analysis

During the protocol stage, the target sample size was set at 293 patients. Based on data from similar patients in our center in 2021–2022, the estimated 28-day mortality rate was approximately 44%, yielding about 129 death events. The main prediction model in the full cohort used multivariable logistic regression, with 8 degrees of freedom of predictors prespecified: age, sex, SOFA score, blood lactate, invasive mechanical ventilation, continuous infusion of vasoactive drugs, tumor stage, and baseline ctDNA〔ln(VAFmax_T0+0.05), linear term〕, with an event/parameter ratio of approximately 16.1. Early dynamic analysis used a 72-h landmark cohort (surviving at 72 h and completing retesting at 72 ± 6 h and passing ctDNA quality control), with the outcome defined as all-cause mortality from 72 h to day 28; ΔctDNA (linear term) was added on the basis of the clinical model, and sample size planning reserved for reductions due to death within 72 h, failure to retest, and testing failures to ensure model stability.

Normality of continuous variables was assessed using the Shapiro–Wilk test: normally distributed variables were expressed as mean ± standard deviation and compared using the t-test, and non-normally distributed variables were expressed as median (interquartile range) and compared using the Mann–Whitney U test; categorical variables were expressed as n (%) and compared using the χ^2^ test, and Fisher’s exact test was used when expected frequencies were <5. Missing data were handled using multiple imputation by chained equations (20 datasets): predictive mean matching for continuous variables, logistic regression for binary variables, and multinomial logistic regression for multicategory variables; the imputation model included the primary outcome, all candidate predictors, and ICU admission time, and regression results across imputed datasets were combined according to Rubin’s rules.

The main association analyses used logistic regression to evaluate the relationship between ctDNA metrics and 28-day mortality, reporting ORs and 95% confidence intervals. Baseline ctDNA was entered as ln(VAFmax+0.05), and dynamic change was entered as ΔctDNA. Nonlinear relationships were assessed using restricted cubic splines (4 knots: the 5th, 35th, 65th, and 95th percentiles). Multivariable adjustment was performed according to prespecified confounders, without stepwise regression; collinearity was assessed using the variance inflation factor, with a threshold of 5.

Prediction models were constructed and evaluated in two steps: first, a clinical baseline model was established (age, sex, SOFA score, blood lactate, invasive mechanical ventilation, continuous infusion of vasoactive drugs, tumor stage), and then baseline ctDNA or dynamic ctDNA was added to form extended models. Discrimination was evaluated using the AUC and its 95% confidence interval, and AUC differences between models were tested using the DeLong test; calibration was evaluated using the calibration intercept, calibration slope, Brier score, and calibration curve. Incremental predictive value was quantified using continuous NRI and IDI, and decision curve analysis was performed (threshold probability 0.10–0.80, step size 0.01). Internal validation used 1,000 bootstrap resamples to calculate optimism-corrected AUC, calibration slope, and Brier score, and regression coefficients were shrinkage-corrected according to a uniform shrinkage factor.

Three prespecified sensitivity analyses were performed: ① repeating the main models using complete-case data; ② rebuilding models by replacing the ctDNA level metric VAFmax with the sum of VAFs of all included somatic variants in the same sample (VAFsum); ③ repeating the dynamic analysis after excluding patients who initiated new anti-tumor treatment (cytotoxic chemotherapy, targeted therapy, immune checkpoint inhibitor therapy, radiotherapy) from baseline to 72 h. Three prespecified subgroup analyses were performed: histologic type (non-small cell lung cancer/small cell lung cancer), whether meeting the definition of sepsis at ICU admission, and whether receiving anti-tumor treatment within 30 days before ICU admission. All tests were two-sided, with α = 0.05; statistical analyses were performed using R (4.4.1).

### Data management

Data were entered and managed using an electronic case report form, and de-identified linkage between clinical data and samples was achieved using a unique study identifier. Two investigators independently verified key variables (primary outcome, ctDNA time points, SOFA, APACHE II, lactate, organ support treatments), and discrepancies were adjudicated by a third investigator through review of the original medical records.

## Results

### Study participants and cohort construction

**FIGURE 1:**
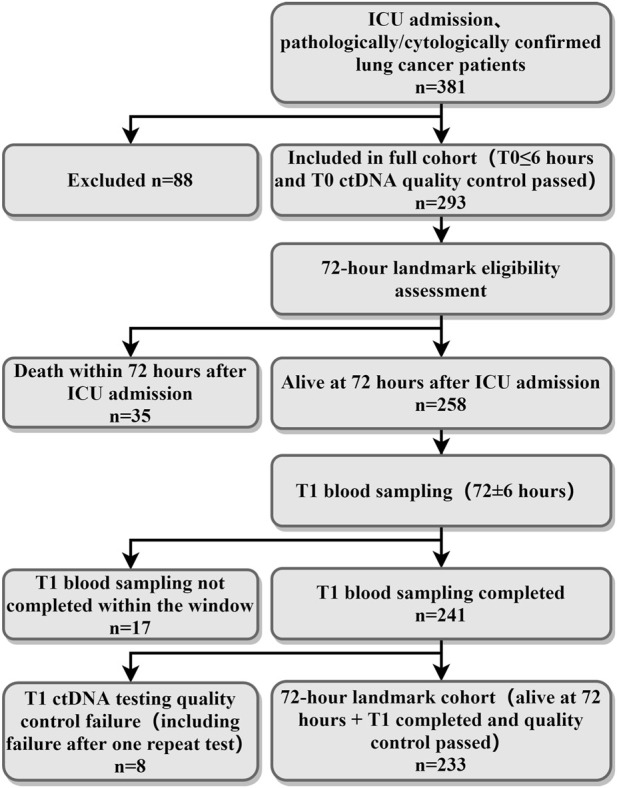
Flow chart of participant screening and construction of the analysis cohort.

During the study period, 381 lung cancer patients were screened in the ICU; 88 were excluded due to stage mismatch, non-first ICU admission, limitation of treatment, or non-compliance with sample/consent requirements, and 293 patients were ultimately included in the full cohort analysis. On this basis, 35 patients died within 72 h; among the remaining 258 patients, 233 completed T1 blood sampling and passed ctDNA quality control, forming the 72-h landmark cohort (Figure 1). Compared with 28-day survivors, non-survivors were older and had higher Charlson comorbidity index and higher ECOG performance status score within 30 days before ICU admission; the proportions of sepsis and septic shock were higher, and disease severity was higher (higher SOFA/APACHE II scores and lactate levels). Non-survivors had worse oxygenation and acid-base status (lower PaO_2_/FiO_2_ and pH), and lower platelet count and albumin. In terms of organ support, invasive mechanical ventilation, continuous infusion of vasoactive drugs, and renal replacement therapy were used more frequently (all P < 0.05) (Table 1).

**TABLE 1:** Baseline clinical and oncologic characteristics of the full cohort.

Variable	Overall (n = 293)	28-day survivors (n = 164)	28-day non-survivors (n = 129)	P
Age (years)	66 [59–73]	63 [55–70]	70 [62–77]	<0.001
Sex (male)	198 (67.58)	105 (64.02)	93 (72.09)	0.181
Body mass index BMI (kg/m^2^)	23.35 [21.45–25.62]	23.35 [21.71–25.62]	23.40 [21.14–25.56]	0.630
Smoking history, n (%)				0.126
Never smoking	112 (38.23)	70 (42.68)	42 (32.56)	
Former smoking	121 (41.30)	66 (40.24)	55 (42.64)	
Current smoking	60 (20.48)	28 (17.07)	32 (24.81)	
Charlson comorbidity index (points)	6 [5–8]	6 [5–7]	7 [6–8]	<0.001
ECOG performance status score within 30 days before ICU admission (points)	2 [1–3]	2 [1–2]	3 [2–3]	<0.001
Histologic type				0.131
Non-small cell lung cancer	252 (86.01)	146 (89.02)	106 (82.17)	
Small cell lung cancer	41 (13.99)	18 (10.98)	23 (17.83)	
Stage				0.117
IIIB	22 (7.51)	16 (9.76)	6 (4.65)	
IIIC	31 (10.58)	20 (12.20)	11 (8.53)	
Stage IV	199 (67.92)	110 (67.07)	89 (68.99)	
Extensive stage (SCLC)	41 (13.99)	18 (10.98)	23 (17.83)	
Brain	78 (26.62)	38 (23.17)	40 (31.01)	0.170
Bone	121 (41.30)	62 (37.80)	59 (45.74)	0.212
Liver	64 (21.84)	28 (17.07)	36 (27.91)	0.037
Adrenal gland	55 (18.77)	26 (15.85)	29 (22.48)	0.197
Pleura	97 (33.11)	49 (29.88)	48 (37.21)	0.231
Anti-tumor treatment within 30 days before ICU admission				0.002
Cytotoxic chemotherapy	78 (26.62)	50 (30.49)	28 (21.71)	
Targeted therapy	62 (21.16)	42 (25.61)	20 (15.50)	
Immune checkpoint inhibitor therapy	41 (13.99)	26 (15.85)	15 (11.63)	
Radiotherapy	29 (9.90)	14 (8.54)	15 (11.63)	
No treatment	83 (28.33)	32 (19.51)	51 (39.53)	
Primary reason for ICU admission				0.004
Acute respiratory failure	156 (53.24)	99 (60.37)	57 (44.19)	
Septic shock	55 (18.77)	19 (11.59)	36 (27.91)	
Non-septic shock	18 (6.14)	8 (4.88)	10 (7.75)	
Severe pulmonary infection without shock	24 (8.19)	17 (10.37)	7 (5.43)	
Acute central nervous system events	12 (4.10)	7 (4.27)	5 (3.88)	
Acute kidney injury requiring renal replacement therapy	9 (3.07)	4 (2.44)	5 (3.88)	
Perioperative monitoring	6 (2.05)	5 (3.05)	1 (0.78)	
Others	13 (4.44)	5 (3.05)	8 (6.20)	
Meeting sepsis within 24 h after ICU admission (yes)	223 (76.11)	106 (64.63)	117 (90.70)	<0.001
Meeting septic shock within 24 h after ICU admission (yes)	87 (29.69)	33 (20.12)	54 (41.86)	<0.001
SOFA score (points)	10 [8–12]	9 [6–10]	12 [10–14]	<0.001
APACHE II score (points)	22 [17–27]	18 [14–22]	27 [23–33]	<0.001
Blood lactate (mmol/L)	2.43 [1.71–3.56]	1.95 [1.32–2.59]	3.18 [2.39–5.26]	<0.001
PaO_2_/FiO_2_ (mmHg)	166 [135–204]	184 [157–225]	141 [105–168]	<0.001
Arterial blood pH	7.33 [7.28–7.38]	7.36 [7.32–7.40]	7.30 [7.25–7.34]	<0.001
Serum creatinine (μmol/L)	113 [75–179]	90 [64–129]	156 [97–244]	<0.001
Total bilirubin (μmol/L)	20.1 [10.8–31.3]	15.1 [8.7–27.0]	25.3 [16.3–37.5]	<0.001
Platelet count (×10^9^/L)	171 [131–218]	195 [157–234]	139 [94–185]	<0.001
INR	1.20 [1.05–1.37]	1.13 [1.03–1.28]	1.29 [1.10–1.55]	<0.001
Serum albumin (g/L)	31.1 [27.3–34.2]	33.0 [30.3–35.9]	28.3 [25.5–31.1]	<0.001
Invasive mechanical ventilation (yes)	171 (58.36)	75 (45.73)	96 (74.42)	<0.001
Non-invasive ventilation (yes)	92 (31.40)	60 (36.59)	32 (24.81)	0.042
High-flow nasal cannula oxygen therapy (yes)	133 (45.39)	84 (51.22)	49 (37.98)	0.032
Continuous infusion of vasoactive drugs (yes)	143 (48.81)	52 (31.71)	91 (70.54)	<0.001
Renal replacement therapy (yes)	47 (16.04)	18 (10.98)	29 (22.48)	0.012

Continuous variables are presented as M [IQR], categorical variables as n (%); SOFA/APACHE II, and laboratory indicators are the worst values within 24 h after ICU, admission; sepsis/septic shock are defined according to Sepsis-3. Two-sided tests were used, with the significance threshold set at α = 0.05.

### Overview of ctDNA samples and testing

A total of 293 samples were sequenced at T0 and all passed quality control; 241 samples were sequenced at T1, of which 233 passed quality control (96.68%). At both time points, total cfDNA amount and effective depth were at a high level; no threshold variants were detected in negative controls, and the pass rate of positive controls was 94.74%. Detectable ctDNA was 83.62% at T0 and 75.54% at T1 (Table 2).

**TABLE 2:** Quality control results for plasma samples and ctDNA testing.

Quality control metrics	T0 (≤6 h)	T1 (72 ± 6 h)
No. sequenced	293	241
Passed	293 (100.00)	233 (96.68)
cfDNA amount (ng)	53.8 [33.1–88.6]	49.6 [30.4–82.9]
Effective depth (×)	11,428 [9276–13964]	10,973 [8842–13311]
On-target rate (%)	74.63 [72.11–78.04]	73.92 [71.36–77.41]
Contamination value (%)	0.23 [0.12–0.39]	0.26 [0.14–0.43]
ctDNA detected	245 (83.62)	176 (75.54)
Negative controls positive	0/19 (0.000)	0/19 (0.000)
Positive controls meeting standard	18/19 (94.74)	18/19 (94.74)

Continuous variables are presented as M [IQR], categorical variables as n (%). Quality control passed: effective depth ≥3,000× and contamination ≤1.0%. Somatic variant threshold: VAF ≥0.10%, with germline/clonal hematopoiesis variants removed.

### Association of Baseline ctDNA and early dynamic changes with 28-day mortality

**FIGURE 2:**
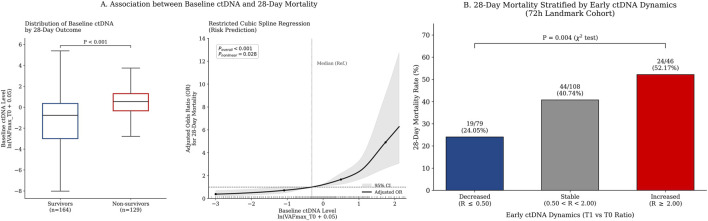
Association of Baseline circulating tumor DNA (ctDNA) and Early ctDNA Dynamics with 28-Day Mortality. **(A)** Association between Baseline ctDNA and 28-Day Mortality. Boxplots of ln(VAFmax, maximum variant allele frequency (VAF),_T0+0.05) in survivors and non-survivors (Mann–Whitney U test). Restricted cubic spline curve of adjusted odds ratios (ORs), using the median baseline ctDNA as reference (OR = 1), adjusted for age, sex, Sequential Organ Failure Assessment (SOFA), lactate, invasive mechanical ventilation, vasoactive drugs, and stage. VAFmax is the maximum VAF in the sample, with non-detection recorded as 0; 0.05 is 1/2 of the detection limit of 0.10%. **(B)** 28-Day Mortality Stratified by Early ctDNA Dynamics. The outcome was all-cause mortality from the 72-h landmark to day 28, and between-group comparisons used the χ^2^ test.

Baseline ctDNA was higher in the non-survivor group than in the survivor group (P < 0.001); adjusted restricted cubic splines showed that ln(VAFmax_T0+0.05) was positively associated with the OR for 28-day mortality and the relationship was nonlinear (P_overall<0.001, P_nonlinear = 0.028) (Figure 2A). In the 72-h landmark cohort, early ctDNA dynamic changes were associated with subsequent 28-day mortality. Compared with those with decreased ctDNA, mortality was higher in those with stable and increased ctDNA (P = 0.004) (Figure 2B). Multivariable logistic regression showed that baseline ctDNA was independently associated with increased 28-day mortality: for each 1-unit increase in ln(VAFmax_T0+0.05), the OR for 28-day mortality was 1.86. In the 72-h landmark cohort, for each 1-unit increase in ΔctDNA, the OR for subsequent mortality was 1.62 (both P < 0.01) (Table 3).

**TABLE 3:** Independent predictive value of baseline ctDNA and ΔctDNA for 28-day mortality.

Analysis cohort	Outcome definition	ctDNA metric	Adjusted OR (95% CI)	P
Full cohort (n = 293)	All-cause mortality from T0 to day 28	ln(VAFmax_T0+0.05)	1.86 (1.48–2.33)	<0.001
72-h landmark cohort (n = 233)	All-cause mortality from 72 h to day 28	ΔctDNA = ln(VAFmax_T1+0.05) − ln(VAFmax_T0+0.05)	1.62 (1.22–2.14)	0.001

Models included the corresponding ctDNA, metric and were adjusted for age, sex; SOFA, score, blood lactate, invasive mechanical ventilation, continuous infusion of vasoactive drugs, and tumor stage. VAFmax, is the maximum VAF in the sample, with non-detection recorded as 0; 0.05 is 1/2 of the detection limit of 0.10%, used for log transformation.

### Prediction model performance and incremental predictive value

**FIGURE 3:**
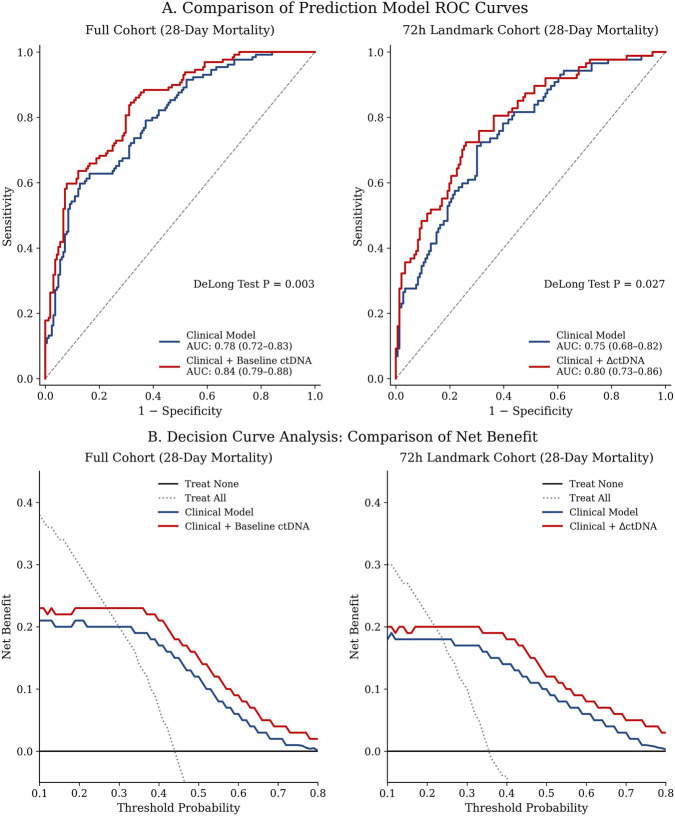
Comparison of Model Discrimination and Clinical Net Benefit. **(A)** Comparison of Prediction Model receiver operating characteristic (ROC) Curves. Clinical model variables: age, sex, Sequential Organ Failure Assessment (SOFA) score, blood lactate, invasive mechanical ventilation, continuous infusion of vasoactive drugs, tumor stage; the extended model added ln(VAFmax, maximum variant allele frequency (VAF),_T0+0.05) or Δcirculating tumor DNA (ctDNA); area under the curve (AUC) differences were tested using the DeLong test. **(B)** Decision Curve Analysis: Comparison of Net Benefit. Net benefit was calculated based on model-predicted probabilities and true outcomes; Treat-all and Treat-none were reference strategies.

In the full cohort, model discrimination improved after adding baseline ctDNA, with the apparent AUC increasing from 0.78 to 0.84; in the 72-h landmark cohort, the AUC increased from 0.75 to 0.80 after adding ΔctDNA (both P < 0.05) (Figure 3A). Overall calibration was good in both cohorts. In the full cohort, after adding baseline ctDNA, the optimism-corrected AUC increased from 0.77 to 0.83 and the Brier score decreased from 0.201 to 0.184; in the 72-h landmark cohort, after adding ΔctDNA, the AUC increased from 0.74 to 0.78 and the Brier score decreased from 0.219 to 0.207, with the calibration intercept close to 0 and the slope slightly below 1 (Table 4). After adding ctDNA, model reclassification and discrimination improved, with continuous NRI and IDI both positive in both cohorts and 95% CIs not crossing 0; NRI/IDI were 0.42/0.08 in the full cohort and 0.29/0.05 in the 72-h landmark cohort (Table 5). Decision curve analysis showed that the ctDNA-extended model outperformed the clinical model in both cohorts; in the full cohort at pt approximately 0.28–0.80 and in the 72-h landmark cohort at pt approximately 0.23–0.80, net benefit was higher than both Treat-all and Treat-none (Figure 3B).

**TABLE 4:** Prediction model calibration, overall performance, and internal validation metrics.

Cohort/Model	Optimism-corrected AUC	Calibration intercept	Calibration slope	Optimism-corrected brier	Shrinkage factor
Full cohort-Clinical	0.77	−0.04	0.96	0.201	0.95
Full cohort-Clinical + baseline ctDNA	0.83	−0.02	0.94	0.184	0.93
72 h landmark-clinical	0.74	0.03	0.91	0.219	0.91
72 h landmark-Clinical + ホ把 tDNA	0.78	0.01	0.89	0.207	0.88

Calibration intercept = 0 and calibration slope = 1 indicate ideal calibration; a smaller Brier score indicates lower overall prediction error. All corrected metrics were obtained based on 1,000 bootstrap resamples.

**TABLE 5:** Assessment of incremental predictive value.

Cohort/Model comparison	NRI (95% CI)	IDI (95% CI)
Full cohort: Clinical vs. Clinical + baseline ctDNA	0.42(0.26–0.61)	0.08(0.04–0.13)
72 h landmark: Clinical vs. Clinical+ΔctDNA	0.29(0.08–0.48)	0.05(0.01–0.09)

Continuous NRI, and IDI, use 0 as the threshold for no incremental value; 95% CIs, were obtained based on 1,000 bootstrap resamples.

### Robustness and sensitivity analyses

**FIGURE 4:**
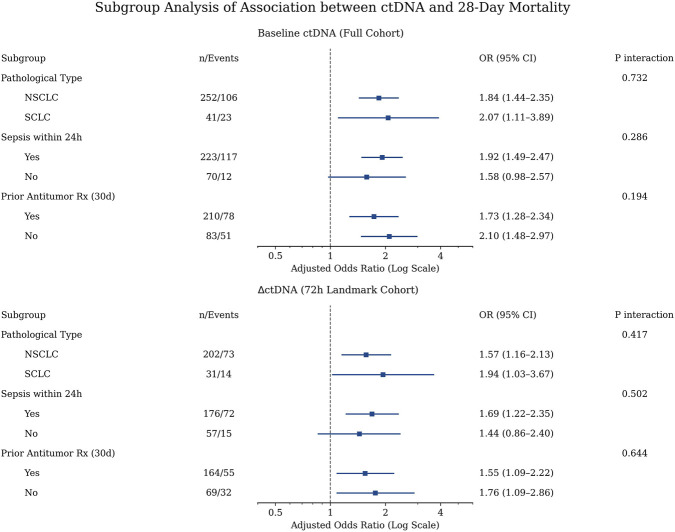
Subgroup Analysis of Association between circulating tumor DNA (ctDNA) and 28-Day Mortality. Odds ratios (ORs) within subgroups were derived from multivariable logistic regression models including interaction terms; P_interaction is the Wald test P value for the interaction term “ctDNA metric × subgroup variable”. Models were adjusted for age, sex, Sequential Organ Failure Assessment (SOFA), lactate, invasive mechanical ventilation, vasoactive drugs, and tumor stage, and included the main effect term for the corresponding subgroup variable.

Prespecified subgroup analyses showed that the effect directions of baseline ctDNA and ΔctDNA were consistent across subgroups (adjusted ORs >1 in all subgroups), and no significant interactions were observed (all P_interaction>0.05) (Figure 4). The proportion of missing key covariates was low, with 14 patients (4.78%) having at least one missing key covariate. Missingness was mainly concentrated in ECOG (9.22%), BMI (6.14%), and types of anti-tumor treatment within 30 days before ICU admission (5.12%), while missing rates for the remaining variables were all ≤4.44%. Multiple imputation was performed according to variable type, and the outcome and ctDNA metrics were not imputed (Table 6). All sensitivity analyses were consistent with the main results; in complete-case analysis, replacing VAFmax with VAFsum, and excluding patients who initiated new anti-tumor treatment within 72 h, the associations between ctDNA metrics and the OR for mortality remained stable (all OR>1 and P < 0.05) (Table 7).

**TABLE 6:** Missingness of key variables and overview of multiple imputation.

Variable	Missing values n (%)	Imputation model type
At least 1 key covariate missing	14 (4.78)	—
SOFA score	3 (1.02)	Predictive mean matching
Blood lactate (mmol/L)	7 (2.39)	Predictive mean matching
Tumor stage (IIIB/IIIC/stage IV/extensive stage)	6 (2.05)	Multinomial logistic regression
Body mass index BMI (kg/m^2^)	18 (6.14)	Predictive mean matching
ECOG performance status score within 30 days before ICU admission	27 (9.22)	Multinomial logistic regression
Charlson comorbidity index (points)	5 (1.71)	Predictive mean matching
APACHE II score (points)	9 (3.07)	Predictive mean matching
PaO_2_/FiO_2_ (mmHg)	13 (4.44)	Predictive mean matching
Arterial blood pH	11 (3.75)	Predictive mean matching
Serum creatinine (μmol/L)	4 (1.37)	Predictive mean matching
Total bilirubin (μmol/L)	5 (1.71)	Predictive mean matching
Platelet count (×10^9^/L)	2 (0.68)	Predictive mean matching
INR	5 (1.71)	Predictive mean matching
Serum albumin (g/L)	8 (2.73)	Predictive mean matching
Types of anti-tumor treatment within 30 days before ICU admission	15 (5.12)	Multinomial logistic regression

This table lists only variables with missing values. Multiple imputation used chained equations to generate 20 imputed datasets; regression analysis results were combined according to Rubin’s rules.

**TABLE 7:** Summary of sensitivity analysis results.

Scenario/Cohort	ctDNA metric	Adjusted OR (95% CI)	P
Complete-case: full cohort (n = 279)	ln(VAFmax_T0+0.05)	1.82 (1.43–2.31)	<0.001
Complete-case: 72 h landmark (n = 223)	ΔctDNA	1.57 (1.17–2.12)	0.003
VAFsum: full cohort (n = 293)	ln(VAFsum_T0+0.05)	1.69 (1.33–2.15)	<0.001
VAFsum: 72 h landmark (n = 233)	ΔctDNA_sum	1.47 (1.10–1.97)	0.009
Excluding new treatment within 72 h: 72 h landmark (n = 207)	ΔctDNA	1.54 (1.11–2.14)	0.01

Adjustment variables were the same as in Table 3; VAFsum, is the sum of VAFs of all included somatic variant sites in the same sample; ΔctDNA_sum = ln(VAFsum_T1+0.05)−ln(VAFsum_T0+0.05). Exclusion criterion: initiation of cytotoxic chemotherapy, targeted therapy, immunotherapy, or radiotherapy from baseline to 72 h.

## Discussion

Baseline ctDNA was markedly higher in 28-day non-survivors, and remained independently associated with an increased OR of mortality even after adjusting for age, sex, SOFA, lactate, invasive mechanical ventilation, vasoactive drugs, and tumor stage, with a nonlinear relationship. This phenomenon suggests that the short-term prognosis of critically ill lung cancer patients is not determined only by the intensity of acute organ failure, and that tumor burden and tumor biological activity can also provide quantifiable incremental information early. ctDNA is released from tumor cell apoptosis and necrosis, and extensive metastatic lesions in advanced disease, as well as hypoxic and inflammatory microenvironments, can accelerate cell turnover, increasing tumor-related fragments in plasma (Stejskal et al., 2023); during the critical illness stage, insufficient hepatic and renal perfusion and inflammation-mediated decreased clearance capacity may amplify differences in ctDNA levels under the same tumor burden (Li et al., 2025). The nonlinear pattern supports the presence of a threshold effect: the low-level range is more affected by the detection limit and random fluctuations, whereas the high-level range may correspond to rapid progression or irreversible depletion of physiological reserve, thereby leading to an accelerated increase in the OR of mortality as ctDNA rises, suggesting that modeling ctDNA as a continuous variable with splines is preferable to simple dichotomous cutoffs (Ma et al., 2023; Lee et al., 2024; Lopez-Ayala et al., 2025). Previous studies in advanced lung cancer have reported associations between baseline ctDNA and tumor burden, treatment response, and long-term survival, but mostly in non-ICU populations with efficacy or long-term outcomes as endpoints (Bartolomucci et al., 2025); studies in critically ill cancer patients have mainly relied on physiological scores such as SOFA/APACHE (Beniwal et al., 2022). Compared with prior studies that mainly focused on non-ICU populations, long-term outcomes, or treatment-response monitoring, the present study is distinguished by its inclusion of critically ill ICU patients with advanced lung cancer, sampling at both ICU baseline and 72 h, and the integrated use of multivariable modeling, restricted cubic splines, NRI/IDI, and decision-curve analysis to evaluate the independent predictive value, reclassification ability, and clinical net benefit of ctDNA. Within the framework of short-term hard outcomes in the ICU, this study verified the independence and nonlinearity of ctDNA, providing evidence closer to the clinical decision window for incorporating tumor-specific molecular signals into early risk stratification.

In the 72-h landmark cohort, early ctDNA dynamic changes could re-stratify patients, with fewer subsequent deaths in patients with decreased ctDNA and worse outcomes in those with stable or increased ctDNA; this association persisted after adjusting for age, SOFA, lactate, organ support treatments, and tumor stage. The landmark unified the outcome starting point at 72 h, reducing time-dependent bias due to the inability to obtain retesting in early deaths. The conclusions remained consistent after excluding patients who initiated new anti-tumor treatment within 72 h, suggesting that the dynamic signal was not triggered only by short-term treatment. ctDNA has a short half-life in plasma, and changes over a 72-h scale are closer to the net effect of tumor cell release and host clearance capacity (Sánchez-Herrero et al., 2022). In critical illness, hypoxia, inflammatory responses, and perfusion abnormalities may accelerate tumor cell turnover and impair hepatic and renal clearance, such that an increase in ctDNA may reflect ongoing release or reduced clearance and is often associated with poorer reversibility of the acute condition. (Koudahl Conrad et al., 2025; Boniface and Spellman, 2022; Cano-Gamez et al., 2025). A decrease in ctDNA is more consistent with reduced tumor-related turnover or recovery of clearance function. This dynamic indicator is similar to the concept of “clearance” in critical care medicine, but the signal comes from tumor-specific mutations and can update prognostic probability while physiological indicators are still fluctuating, providing time-sensitive prognostic information for monitoring intensity and care communication (Pan et al., 2019). Previous lung cancer studies have mostly evaluated the consistency between ctDNA decreases and radiographic remission and survival benefit on a scale of weeks in targeted therapy or immunotherapy settings (Anagnostou et al., 2023). The present results translate this logic to the ICU short-term mortality framework, supporting that ctDNA dynamics are not only used for efficacy monitoring but can also serve as a prognostic re-stratification variable during the critical illness stage.

The clinical model consisted of age, sex, SOFA, lactate, organ support, and stage; discrimination improved after adding baseline ctDNA or 72-h ΔctDNA, and the advantage remained after internal validation, indicating that ctDNA provided incremental information independent of organ failure indicators. The extended model had a calibration intercept close to 0 and a slope close to 1, with lower overall error, and the shrinkage factor suggested controllable overfitting; predicted probabilities were more reliable and these findings should be interpreted cautiously for individual risk assessment. The confidence intervals of continuous NRI and IDI did not cross 0, supporting improved individual reclassification. Decision curve analysis showed that the ctDNA-extended model had higher net benefit than the clinical model, and was higher than both Treat-all and Treat-none strategies across a wide threshold range, suggesting exploratory potential for prognostic risk stratification pending external validation, but it cannot be used to infer that ctDNA-based interventions can improve outcomes. Mechanistically, clinical variables mainly characterize acute physiological imbalance, whereas ctDNA characterizes tumor burden and biological activity; these two types of signals have different sources, and joint modeling can better reflect heterogeneity in short-term mortality probability (Assaf et al., 2023). This complementarity explains the consistent performance improvement in both the full cohort and the landmark cohort; ctDNA may have potential value for risk stratification at ICU admission and for risk updating at 72 h, pending external validation (Putter and van Houwelingen, 2022). With stable testing quality control, low covariate missingness, and standardized imputation procedures, with no imputation for outcomes and ctDNA metrics, and consistent sensitivity analysis conclusions, the credibility of the model’s incremental value was strengthened. Previous tumor biomarker studies have mostly focused on AUC improvement, with fewer reporting calibration, internal validation, and net benefit simultaneously (Dhiman et al., 2022). These key dimensions were supplemented in the ICU setting, making the results more amenable to peer review and interpretation.

This study was a prospective single-center cohort, and the enrollment source, referral structure, and ICU care pathways were center-specific; whether the model maintains the same discrimination and calibration in other hospitals with different patient case-mix, testing workflows, and treatment intensity remains unknown and requires external validation. Local treatment strategies, baseline patient characteristics, and testing workflow differences may all affect model transportability; future multicenter studies across different testing platforms and analytical pipelines are needed to further validate discrimination, calibration, and transportability. Some patients were not enrolled due to failure to complete blood sampling within the specified time, unqualified samples, or failure to obtain informed consent, which may introduce selection bias. The dynamic analysis used a 72-h landmark, which can reduce time-dependent bias, but excluded deaths within 72 h and those who did not complete retesting; therefore, the conclusions mainly apply to patients who survived at 72 h and had qualified T1 testing, and only two sampling time points (T0 and T1) were used, making it impossible to evaluate the information gain from earlier or more frequent monitoring. ctDNA testing relied on a fixed panel and quality control thresholds, and exposure variables were defined by VAFmax and its log transformation; because VAFmax is driven by the single highest-VAF site, it may be sensitive to outlying high-VAF variants and may not fully capture overall tumor burden or clonal heterogeneity; as a relative metric, VAF may theoretically be affected by fluctuations in background total cfDNA, and given the high prevalence of sepsis in this cohort, changes in wild-type cfDNA during severe infection and its treatment may influence the biological interpretation of VAF and its early dynamics. In addition, standard K2-EDTA tubes rather than specialized cfDNA preservation tubes were used; despite plasma separation within 2 h, residual leukocyte lysis and background wild-type DNA contamination cannot be completely excluded. Values may not be directly comparable across platforms, different panels, or different quantification methods. Because this study did not further use absolute mutant copies per milliliter of plasma or adjust for total cfDNA levels, the findings are better interpreted as the prognostic value of a relative ctDNA signal rather than a direct quantification of absolute tumor burden change. Although tumor stage was included and histologic type, metastatic sites, and comorbidity data were collected, comorbidities, histologic differences, and incompletely quantified metastatic burden may still influence ctDNA levels and contribute to residual confounding; the absence of imaging-based tumor size also limits further interpretation of the tumor-burden dimension. This study also did not include conventional serum protein tumor markers; therefore, the relative value of ctDNA versus traditional markers in predictive performance, turnaround time, and cost could not be assessed. Formal economic evaluation was not performed in this study; future clinical translation of ctDNA will also require assessment of turnaround time, platform consistency, and cost-effectiveness. The study used 28-day all-cause mortality as the endpoint and did not assess longer-term survival and functional outcomes. The study was predictive and ctDNA results were not returned to clinicians; it did not evaluate whether ctDNA-driven treatment or care decisions could improve outcomes, the observational design cannot fully exclude residual confounding, and imputation of missing data based on model assumptions may also affect estimates.

## Conclusion

Baseline ctDNA was independently associated with 28-day mortality in critically ill ICU patients with advanced lung cancer, showing a nonlinear exposure–response relationship; 72-h ΔctDNA was associated with the disease-course trajectory after admission and enabled risk re-stratification within the 72-h landmark population. After incorporating ctDNA into a clinical model that included SOFA, lactate, and organ support indicators, discrimination, calibration, and reclassification performance were modestly improved, and decision curve analysis suggested higher net benefit across the evaluated threshold probability range. ctDNA testing and quality control were feasible in the ICU workflow and may have potential value for short-term prognostic stratification pending external validation. The study was positioned as predictive and does not support causal inference regarding ctDNA-guided intervention effects; broader application requires external validation in other populations.

## Data Availability

The datasets presented in this article are not readily available because of institutional ethical requirements, patient privacy protection obligations, and applicable regulations on human genetic resource data. Requests to access the de-identified clinical dataset should be directed to the corresponding author.
